# Metabolomics Analysis of Rabbit Plasma after Ocular Exposure to Vapors of Sulfur Mustard

**DOI:** 10.3390/metabo14070349

**Published:** 2024-06-21

**Authors:** Jihéne Bouhlel, Fanny Caffin, Fanny Gros-Désormeaux, Thierry Douki, Jean-François Benoist, Florence A. Castelli, Emeline Chu-Van, Christophe Piérard, Christophe Junot, François Fenaille

**Affiliations:** 1 CEA, INRAE, Département Médicaments et Technologies pour la Santé (DMTS), SPI, MetaboHUB-IDF, Université Paris-Saclay, 91191 Gif-sur-Yvette, France; jihene.bouhlel@hotmail.fr (J.B.); jean-francois.benoist@aphp.fr (J.-F.B.); florence.castelli@cea.fr (F.A.C.); emeline.chu-van@cea.fr (E.C.-V.); christophe.junot@cea.fr (C.J.); 2 Institut de Recherche Biomédicale des Armées (IRBA), 91223 Brétigny-sur-Orge, France; fanny.caffin@def.gouv.fr (F.C.); fanny.gros-desormeaux@def.gouv.fr (F.G.-D.);; 3 CEA, CNRS, IRIG, SyMMES, Université Grenoble Alpes, 38000 Grenoble, France; thierry.douki@cea.fr; 4 Biochemistry Laboratory, APHP, Hôpital Universitaire Necker Enfants Malades, 75015 Paris, France

**Keywords:** sulfur mustard, eyes, rabbits, metabolomics, high-resolution mass spectrometry, SM adducts, biomarkers, plasma

## Abstract

Sulfur mustard (SM) is a highly potent alkylating vesicant agent and remains a relevant threat to both civilians and military personnel. The eyes are the most sensitive organ after airborne SM exposure, causing ocular injuries with no antidote or specific therapeutics available. In order to identify relevant biomarkers and to obtain a deeper understanding of the underlying biochemical events, we performed an untargeted metabolomics analysis using liquid chromatography coupled to high-resolution mass spectrometry of plasma samples from New Zealand white rabbits ocularly exposed to vapors of SM. Metabolic profiles (332 unique metabolites) from SM-exposed (n = 16) and unexposed rabbits (n = 8) were compared at different time intervals from 1 to 28 days. The observed time-dependent changes in metabolic profiles highlighted the profound dysregulation of the sulfur amino acids, the phenylalanine, the tyrosine and tryptophan pathway, and the polyamine and purine biosynthesis, which could reflect antioxidant and anti-inflammatory activities. Taurine and 3,4-dihydroxy-phenylalanine (Dopa) seem to be specifically related to SM exposure and correspond well with the different phases of ocular damage, while the dysregulation of adenosine, polyamines, and acylcarnitines might be related to ocular neovascularization. Additionally, neither cysteine, N-acetylcysteine, or guanine SM adducts were detected in the plasma of exposed rabbits at any time point. Overall, our study provides an unprecedented view of the plasma metabolic changes post-SM ocular exposure, which may open up the development of potential new treatment strategies.

## 1. Introduction

Sulfur mustard (SM; C_4_H_8_Cl_2_S) or bis-(2-chloroethyl)-sulfide, also known as yperite, is a chemical vesicant warfare agent that was widely used during World War I (1914–1918) [1]. Despite its prohibition by the Organization for the Prohibition of Chemical Weapons, it has been recently used in Syria and Iraq in 2015–2016 [2]. Because of its ease of production, its insidious nature, and the lack of a specific antidote, SM has been used as a chemical weapon agent during military conflicts but also during terrorist attacks, while the medical management of SM exposure is still symptomatic [3]. Moreover, there is a high risk of accidental exposure, since numerous unused SM-containing munitions are still present worldwide [4]. Although the toxic effects of SM have been largely documented, its precise mechanism of action remains quite elusive. The routes of exposure to SM are primarily the eyes, skin, and the respiratory system via the inhalation of toxic fumes [5]. The eyes are the most sensitive organs after SM airborne exposure, but ocular injuries have only been sporadically studied at least from a mechanistic point of view [6]. In addition to its acute toxicity, victims can develop chronic eye pathologies even years after their exposure [7,8]. The clinical effects of SM exposure are often characterized by an initial asymptomatic latent period of 6–24 h before the development of pain and lesions ranging from mild conjunctivitis to incapacitating corneal lesions [9,10]. While many affected individuals re-cover almost completely, others can present with delayed and potentially acute toxicity [11].

SM is rapidly hydrolyzed after absorption to a reactive episulfonium ion that can al-kylate the nitrogen, oxygen, and sulfur nucleophilic groups and can crosslink DNA or other biological macromolecules [12,13,14], thus potentially causing genotoxic and cytotoxic injuries [15,16,17]. In this context, the detection of hydrolysis and oxidation products, and/or adducts to biomolecules, in human biofluids or tissues appears relevant as biomarkers of SM exposure [18]. SM-induced genotoxicity was previously reported with the detection of several DNA adducts—N7-hydroxyethylthioethyl-guanine (HETE-N7Gua) and N3-hydroxyethylthioethyl-adenine (HETE-N3Ade)—and the intra- and inter-strand crosslink between two guanines bis(N7-guanyl)-ethylthioethyl (N7Gua-ETE-N7Gua) in the skin [19] and in the organs [13] of mice following cutaneous exposure. Moreover, the toxicity of SM was widely explained by the depletion of glutathione (GSH), which appears as a major target of SM in exposed tissues, inducing oxidative stress via the reduction in cellular antioxidant capacities. Evidence for the occurrence of this pathway in humans is shown through the detection of bis-mercapturate adducts of mustard sulfone in the urine of exposed victims [20]. Based on the known metabolism pathway of GSH conjugates to xenobiotics, other derivatives are expected to be produced, including the cysteine conjugate (Cys-SM) and the final product of the mercapturate pathway N-acetyl-cysteine conjugate (NAC-SM). Similar compounds were actually detected in the plasma of mice exposed to the monofunctional simulant (2-chloroethyl)-ethyl-sulfide (CEES) [21]. Other effects including oxidative stress, inflammation, and severe immune reactions were described after SM intoxication [22,23]. Despite the research progress made for the characterization of SM effects, the mechanism of action remains poorly understood [24]. In that context, metabolomics appears as a powerful branch of omics, which can be employed for studying an organism’s response to external stressors such as disease [25,26,27] or xenobiotic exposure [28,29]. It seeks to analyze the whole set of endogenous and exogenous low molecular mass metabolites present in cells, tissues, or biofluids of an organism. Untargeted metabolomics is generally conducted using Nuclear Magnetic Resonance (NMR) or more often using liquid chromatography coupled to high-resolution mass spectrometry (LC–HRMS). Very few metabolomics studies have explored the metabolic changes occurring in biofluids following SM exposure. For instance, the downregulation of vitamin B6 and tryptophan-derived metabolites has been shown using ^1^H-NMR, while bile acid metabolism proved to be upregulated in the serum of veterans exposed to SM and suffering from chronic complications [30]. ^1^H-NMR metabolomics analysis of plasma samples from subcutaneously treated rats with SM showed disordered metabolism pathways of ketone bodies synthesis, arginine and proline metabolism, butanoate metabolism, and alanine aspartate and glutamate metabolism 24h post-exposure [23].

More recently, LC–HRMS-based untargeted metabolomics pointed out the significant dysregulation of arginine and proline metabolism, histidine metabolism, purine metabolism, and primary bile acids synthesis in the plasma of topically treated mice with CEES several days post-exposure [31]. To the best of our knowledge, metabolomics has not yet been used to explore the systemic changes occurring in the blood of individuals or animal models following ocular exposure to SM.

Rabbits are considered as a relevant animal model for this objective since they have anatomical and physiological features adapted for ocular research and a greater similarity to human eyes than other laboratory animals such as mice or rats [32]. In the present study, we performed an untargeted metabolomics approach based on the LC–HRMS analysis of plasma samples from rabbits ocularly exposed to vapors of SM to study the metabolic pathways that are mostly impaired by sulfur mustard ocular exposure, to obtain a better understanding of the molecular mechanism of SM toxicity and to highlight potential valuable biomarker candidates. In parallel, we also specifically monitored the plasmatic levels of SM-conjugates with cysteine (Cyc-SM), N-acetylcysteine (NAC-SM), and guanine (Gua-SM) as biomarkers of exposure to SM using targeted UHPLC–MS/MS.

## 2. Materials and Methods

### 2.1. Animal Experiments

**Figure 1:**
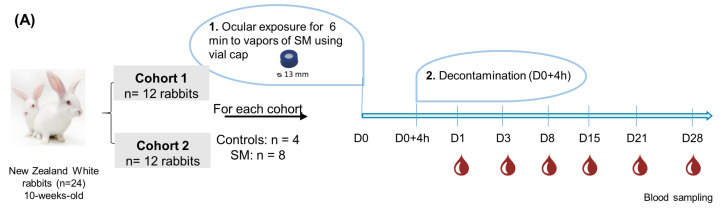

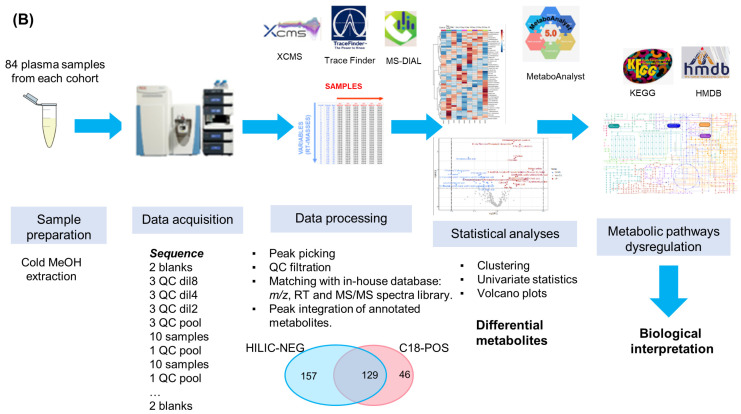
Study overview. (**A**) Experimental design and protocol of rabbit ocular exposure to sulfur mustard (SM); D indicates day. (**B**) Untargeted metabolomics workflow based on LC–HRMS analysis of plasma samples from control and SM-exposed rabbits in both cohorts.

This work has been performed in accordance with the Directives of the European Communities Council Directive 2010/63/UE and was submitted to and approved by the ethical committee of the French Service de Santé des Armées (CEA-SSA). Animal experiments were conducted over two consecutive years and plasma were analyzed in two sessions (the first and second analysis sessions were noted hereafter as cohort 1 and cohort 2). In each cohort, twelve 10-week-old female New Zealand white rabbits weighing 2.5–3.0 kg were received and housed individually. The principles of laboratory animal care were followed in all experiments. The animals were monitored daily to ensure their well-being and they were provided a standard diet and water ad libitum. An environmental light cycle was set up with 16 h of light and 8 h of darkness per day, with a maximum light intensity of 40 lux to reduce photophobia. Rabbits were subjected to 1 week of quarantine followed by 2 weeks of habituation before SM exposure. Exposures were performed in the toxicology laboratories of the French “Institut de Recherche Biomédical des Armées (IRBA)”. SM was supplied by the French “Direction Générale de l’Armement (DGA)”. Handling precautions included the use of personal protective equipment and handling under a chemical fume hood. For each cohort, the animals were randomly divided into the following two groups: unexposed (control group; n = 4) and exposed animals (SM group; n = 8). Rabbits were anesthetized via an intramuscular (i.m.) injection of a mixture of ketamine/medetomidine (15.0 mg/kg; 0.25 mg/kg), and then buprenorphine (0.02 mg/kg) was administered subcutaneously (s.c.) to the animals. A buprenorphine delivery pump was also implanted aseptically in the middle of the scapula to deliver a continuous and constant infusion of pain control medication (Alzet osmotic pump, Charles River Laboratories France, model 2 mL 1–10, 10 μL/h, 7 days). For the SM vapor exposure, 10 μL of neat SM were deposited on a paper filter placed into a 13 mm vial cap whose diameter perfectly fits with the rabbit cornea (~12 mm). Caps were then returned and fixed on the right rabbit eye for a duration of 6 min, while a control cap was fixed in the same way on the left cornea as an internal control. Of note, caps were fixed on the rabbit eye using an ophthalmic gel (Ocry-Gel, TVM Lab, Lempdes, France), thereby avoiding the lateral diffusion of SM. Eyes were then decontaminated 4 h post-exposure using physiological serum. For euthanasia at day 35, rabbits were firstly anesthetized using a mixture of ketamine and medetomidine (15.0 mg/kg; 0.25 mg/kg, i.m.) and were then euthanized via the administration of a lethal intracardiac injection of pentabarbital (333 mg/kg). Then, 30 min prior to blood sampling, lidocaine/prilocaine cream was applied. Blood samples were collected into lithium heparin tubes from the ear marginal vein at 1, 3, 8, 15, 21, and 28 days after exposure (Figure 1A). Blood plasma were isolated using centrifugation (2000 *g*, 10 min, 4 °C) and were then stored at −80 °C.

### 2.2. Untargeted LC–HRMS Metabolomics

For untargeted metabolomics analysis, sample preparation of plasma was performed according to a protocol previously developed and used routinely in our team [33,34]. Two 50 μL aliquots of each plasma sample were extracted separately. Briefly, each aliquot was mixed with 200 μL of cold methanol (MeOH) containing a mixture of internal standards (Table S1). Plasma samples were incubated on ice for 90 min to allow for complete protein precipitation and were then centrifuged (20,000 *g*, 10 min, 4 °C). The supernatants containing metabolites were pipetted off and dried under a nitrogen stream at 30 °C. Each sample was resuspended in 150 μL of the mobile phases used for detection in ESI negative and positive modes and spiked with external standards (Table S1). Quality control (QC) samples were obtained by pooling 10 μL of each sample and were separately extracted. QC samples were injected every 10 samples throughout the analysis to assess sample repeatability and stability, while diluted QC samples (1/2, 1/4 and 1/8) were injected in triplicate at the beginning of the sequence for further data processing (Figure 1B).

Plasma extracts were analyzed using LC–HRMS using the Ultimate 3000 liquid chromatographic system coupled to the Q-Exactive mass spectrometer from Thermo Fisher Scientific (Courtaboeuf, France). The chromatographic separation of metabolites was performed on a ZIC-pHILIC hydrophilic interaction column (SeQuant ZIC -pHILIC column, 5 μm, 2.1 × 150 mm, Merck, Darmstadt, Germany) and a C18 reversed-phase column (Hypersil Gold C18, 2.1 × 150 mm, 1.9 μm, Thermo Fisher Scientific, Bremen, Germany) using ESI negative and positive modes, respectively (named HILIC-neg and C18-pos hereafter). The elution gradients of the mobile phases and mass spectrometry parameters of negative and positive modes were previously detailed in our publications [33,34].

### 2.3. UHPLC–MS/MS Targeted Quantification of SM Adducts

The assay previously designed for the quantification of the biomarkers of 2-chloroethyl ethyl sulfide in plasma samples [21] was presently extended to sulfur mustard derivatives. The standards of the targeted compounds, 2-hydroxyethyl-thioethyl-S-cysteine (Cys-SM), 2-hydroxyethyl-thioethyl-S-N-acetylcysteine (NAC-SM), and 2-hydroxyethyl-thioethyl-N7-guanine (Gua-SM), were prepared through the incubation of 10 mg of cysteine, N-acetylcysteine, and 2′-deoxyguanosine, respectively, with 2-chloroethyl 2-hydroxyethyl sulfide (ChemSpace, Riga, Letonia) in 5 mL of 10 times diluted PBS. The guanine adduct was obtained via the thermal depurination of the 2-deoxyguanosine derivative. Adducts of interest were isolated using reverse-phase HPLC and were characterized using mass spectrometry. Isotopically labeled internal standards were synthesized using the same protocol with either [^13^C_3_,^15^N]-cysteine, [^13^C_3_,^15^N]-N-acetylcysteine, or [^15^N_5_]-2′-deoxyguanosine. Each plasma sample (50 μL) was diluted in 50 µL of water and was spiked with internal standards (1 μL, 10 μM). The samples were loaded onto a 0.2 μm filtration column (VWR) and were centrifuged at 10,000 *g* for 5 min. The filtrate was collected and loaded onto a 30 kDa Nanosep^®^ tube. This second filtration step involved a centrifugation for 20 min at 10,000 *g*. Additional sample clean-up was performed directly on-line within the UHPLC system. The samples were injected (50 μL) first in a Macherey-Nagel Nucleodur^®^ PFP column (50 mm × 2.0 mm ID, 5 μm particle size) used as an on-line SPE column. Mobile phase A consisted of 5 mM ammonium formate (AmF) in MilliQ water, and mobile phase B was methanol with 5 mM AmF. Samples were cleaned using a gradient from 0 to 50% of B in 1 min. The flow rate was set to 350 μL/min, the column temperature was maintained at 50 °C, and the sample storage compartment was set to 15 °C. After that, samples were injected by backflush into the analytical column [31]. Detection was performed using a 6500+ QTrap system (Sciex, Darmstadt, Germany) operated in the “multiple reaction monitoring mode (MRM)”. For each analyte and corresponding internal standard, an MRM transition was used for quantification, together with two qualifying signals.

### 2.4. Data Processing and Analysis of Metabolomics Data

Data processing was conducted on the Workflow4Metabolomics (W4M) platform [35]. LC–HRMS raw files were first converted into mzXML files using the MSConvert software. XCMS (version 2.1) parameters were adapted for peak picking, peak grouping, and time alignment. Then, automatic peak detection and integration were performed, which returned a data matrix containing each *m*/*z* and retention time (RT) values of features, together with the corresponding chromatographic peak areas. Robust metabolic features were retrieved following a three-step filtration process, as follows: (i) ratio of chromatographic peak areas obtained for biological to blank samples >3, (ii) coefficient of variation (CV) of metabolites in the QC samples <30%, and (iii) correlation between QC dilution factors and areas of chromatographic peaks >70%. Metabolite annotation was performed thanks to an in-house chemical database by matching accurate measured masses (<10 ppm) and chromatographic RT to those of more than 1200 pure authentic standards analyzed under identical conditions [33]. Metabolite identification was achieved by running additional LC–HRMS/MS analyses on QC samples. Obtained mass spectra were matched both manually and using the MS-DIAL 4.60 software to the spectra included in our in-house spectral database, as described elsewhere [36] (Table S2). To ensure accurate and consistent metabolite quantification, the chromatographic peaks of the annotated metabolites were integrated for all the samples using the TraceFinder 4.1 SP1 software (Thermo Fisher Scientific) and the resulting peak integrations were manually reviewed.

### 2.5. Statistical Analyses

From each cohort, the data matrix of annotated metabolites was log10 transformed. Statistical analysis was conducted using MetaboAnalyst 5.0 (http://www.metaboanalyst.ca, accessed on 8 December 2023). Firstly, hierarchical clustering analysis was carried out on the control group (n = 4) and the SM group (n = 8) at different time points using Pearson’s correlation on the top 50 most significant features selected using one-way Analysis of Variance (ANOVA; *p*-values < 0.05). Secondly, Volcano plots were elaborated on data from control group vs. SM-exposed group at each time point using a non-parametric Wilcoxon Mann–Whitney test (*p*-values < 0.05). Metabolites with *p*-values < 0.05 and fold changes (FCs) < 0.8 or >1.2 were selected as the most relevant ones. After the significant metabolites were selected, the related biochemical pathways were established using the Kyoto Encyclopedia of Genes and Genomes (KEGG) database, the Human Metabolome database (HMDB), and previous studies.

## 3. Results and Discussion

In the present study, animal experiments were performed as two independent cohorts (hereafter referred to as cohort 1 and cohort 2). In each cohort, twelve ten-week-old female New Zealand white rabbits were randomly divided into two groups—unexposed (control group; n = 4) and exposed animals (SM group; n = 8) (Figure 1A). Rabbits were ocularly exposed to SM vapor for 6 min, using caps to limit exposure to the cornea and to avoid direct chemical injury of the ocular adnexa [32]. For both cohorts, blood samples were collected from the control and exposed rabbits at 1, 3, 8, 15, 21, and 28 days after SM exposure for further metabolomics analysis (Figure 1A), while clinical measurements were used to assess the ocular toxicity of SM over time. As described by Caffin et al. (in preparation), animals exposed to SM expressed clinical symptoms such as a transient increase then a decrease in Intraocular Pressure (IOP), corneal opacity, the apparition of neovascularization (Figure S1), an increase in cornea thickness, and a deformation of the eyeball.

### 3.1. Plasma Metabolite Profiling of Control and SM-Exposed Rabbits

Untargeted metabolomic analysis was performed on the plasma samples collected during the first and second independent cohorts to detect potential changes in plasma metabolic profiles following ocular exposure to SM and to obtain a deeper insight into the biochemical mechanisms underlying SM toxicity (Figure 1B). LC–HRMS analyses were performed using two complementary chromatographic conditions involving HILIC with detection in the negative ionization mode (designated hereafter as HILIC-Neg) and C18 in positive ionization mode (C18-Pos), allowing for the analysis of polar and more hydrophobic metabolites, respectively [33,34]. Up to 191 metabolite features from the HILIC-Neg and 176 from the C18-Pos datasets matched the accurate *m*/*z*, RT, and the MS/MS spectra of pure standards included in our in-house library, resulting in 332 unique metabolites annotated in both cohorts (Table S2). These annotated metabolites belong to different chemical families, such as amino acids, carbohydrates, lipids, nucleosides, nucleotides, and organic acids.

### 3.2. Global Metabolic Changes Occurring in Plasma Post-SM Exposure

Importantly and for both cohorts, the 332 metabolites monitored in the plasma of control rabbits showed no significant differences from day 1 to day 28, demonstrating the absence of any significant effects related to development or age-related effects during this particular time window.

**Figure 2:**
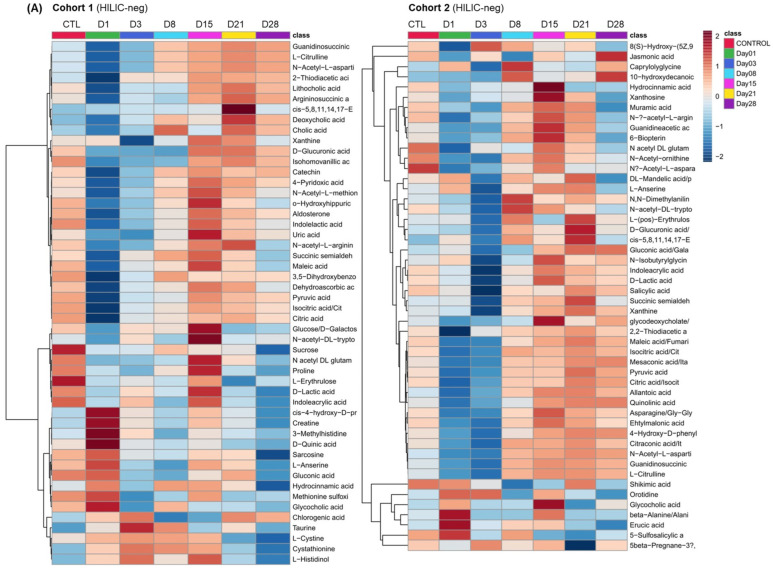

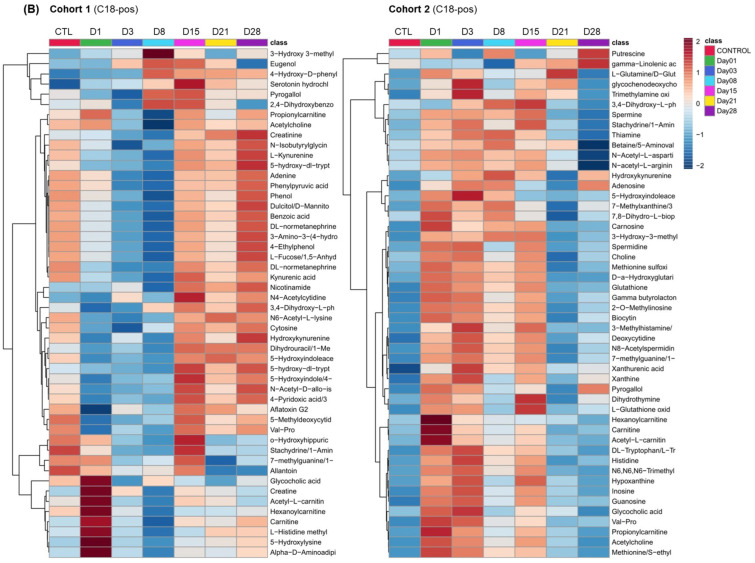
Heatmap analysis of the metabolomics profiling in cohorts 1 and 2, based on the first 50 significant metabolites selected using ANOVA (*p* ≤ 0.05) between the control group (average from day 1 to day 28) and the SM-exposed group from day 1 to day 28. The heatmap indicates high (red) and low (blue) mean relative abundances (log-transformed) of metabolites using (**A**) HILIC-negative ionization datasets and (**B**) C18-positive datasets.

To obtain a global overview of the kinetic metabolic changes occurring in the plasma of SM-exposed rabbits, multivariate data treatment was first performed using hierarchical clustering analysis on the HILIC-Neg and C18-Pos data matrices separately, based on the top 50 significant metabolites selected using ANOVA (*p* < 0.05) for the two cohorts (Figure 2). Heatmap analysis of the HILIC-Neg and C18-Pos data highlighted a time-dependent evolution of metabolite patterns over the 28 days post-exposure (Figure 2). Slight discrepancies in the time-dependent evolution of some metabolites can be observed in the two cohorts and could be the consequence of some inter-individual variability with more/less intense or potentially delayed responses of the rabbits after exposure to SM. Also, the dose of SM to which the rabbits were exposed might slightly vary if the SM vapor dispensing cup was not perfectly installed on the eye of the rabbit [37]. Although not possible to implement in the frame of this study, increasing rabbit numbers can alleviate some of these issues.

Thus, despite some inter-individual variability, we noted the early accumulation of some metabolites belonging to the amino acid (e.g., histidine, methionine, and cysteine), carnitine, and creatine metabolic pathways on days 1 and 3 in both cohorts. These metabolic changes are potentially representative of a systemic inflammation associated with an alteration of the energy metabolism. The dysregulation of the urea cycle, purines, and the TCA cycle metabolites was then observed at day 15 and beyond.

These time-dependent changes in plasma metabolite profiles are consistent with the different phases of ocular damages following SM exposure. Interestingly, ocular lesions started at 24 h post-exposure, followed by spontaneous healing between 72 h and 96 h up to a week post-exposure, while a delayed phase of pathological processes was observed as early as 2 weeks post-exposure to SM [38]. Our data corroborated previous studies reporting a reliable pattern of ocular manifestations of keratopathy after exposure to SM, starting with an acute phase lasting 1–2 weeks, followed by a latent phase in which clinical signs re-appear several weeks after resolution, and a delayed phase in which signs re-appear 3–5 weeks after exposure [7,32,37].

To encompass the observed variability in the rabbit response to SM ocular exposure, we pursued our investigations by focusing on metabolites that showed a similar behavior in both cohorts, first to fish out potential specific effect biomarkers and then to provide preliminary insights into the biochemistry underlying SM toxicity.

### 3.3. Looking for Biomarkers of SM Exposure

In order to better characterize the systemic metabolomics changes occurring in plasma after ocular exposure to SM, we performed a univariate statistical comparison between the exposed and control groups at each time point using Volcano plots based on the non-parametric Wilcoxon Mann–Whitney test (*p* < 0.05). From the list of 332 annotated metabolites in both cohorts, 91 and 108 proved to be significantly different with fold-changes (FC) < 0.8 or >1.2 in the SM-exposed rabbits compared to the controls at any time point in cohorts 1 and 2, respectively, while 33 shared behaviors between both cohorts (Table S3). Among these, ten showed similar metabolic time-dependent dysregulation throughout the 28 days post-exposure and were therefore considered as potentially relevant biomarker candidates (Table 1).

Most striking is the upregulation of taurine from day 3 to day 15 that is consistently observed in both cohorts after SM exposure, which can indicate the activation of antioxidant and anti-inflammatory responses (Table 1). Taurine, a sulfonic amino acid in which the carboxylic group is replaced by a sulfonic acid group, is one of the most abundant amino acids in the retina, cornea, and lens, as well as in the vitreous and aqueous humors [39]. Taurine has also been described as a promising metabolite biomarker in several ocular pathologies, essentially due to its multiple functions in osmoregulation, antioxidant defense, anti-inflammatory response, protein stabilization, neuro-modulation, and immuno-modulation, thereby contributing to cell protection against various types of injury [40,41]. Interestingly, high plasma levels of taurine were found in affected dogs with glaucoma, which was attributed to a compensatory mechanism counteracting oxidative stress [42].

In addition, the data also pointed out an accumulation of 3,4-dihydroxy-phenylalanine (Dopa) during the first 8 days (Table 1). Dopa is a neurotransmitter considered to be redox active with antioxidant properties against oxygen radicals [43]. Dopa has also been reported to inhibit DNA cleavage and low-density lipoprotein oxidation [44], while the primary sites of SM damages were DNA, protein, and lipid modifications [45]. To a lesser extent, an accumulation of 5-hydroxyindoleacetic acid, 3-hydroxy-3-methylbutyric acid, and adenosine was observed, while a downregulation of allantoic acid, N-acetyl-neuraminic acid, N-acetyl-glutamic, and isocitric acid/citric acid was also observed during the first two weeks post-exposure (Table 1). Later on, increased levels of eicosapentaenoic acid were detected during the delayed phase of SM damages (between day 21 and day 28) (Table 1). Overall, these candidate biomarkers, essentially taurine and Dopa, seem to be specifically related to rabbit exposure to SM and correspond well with the different phases of ocular damage. Of course, this would warrant further investigation to confirm the relevance of the biomarkers.

### 3.4. Metabolic Pathways Dysregulated after Ocular Exposure to SM

We investigated the significant plasma metabolites modulated within metabolic pathways based on pathway analysis to better understand the metabolic effects in plasma after ocular exposure to SM. The most impacted pathways were the glycolysis, the TCA cycle, the amino acids metabolism, the purine and polyamine biosynthesis, and the phenylalanine, tyrosine, and tryptophan metabolism (Table 1). We also noted significant metabolites that were modulated differently in both cohorts. The relevance of the mechanisms that induced changes in these metabolic pathways is a critical step toward understanding SM toxicity following ocular exposure and revealing promising candidate biomarkers for further therapeutic purposes.

#### 3.4.1. Ocular Exposure to SM Induces Systemic Inflammation

The perturbation of energy metabolism, amino acid metabolism, and purine and polyamine biosynthesis was directly related to oxidative stress resulting from an imbalance between reactive oxygen species (ROS) formation and their neutralization by cellular antioxidants.

**Figure 3:**
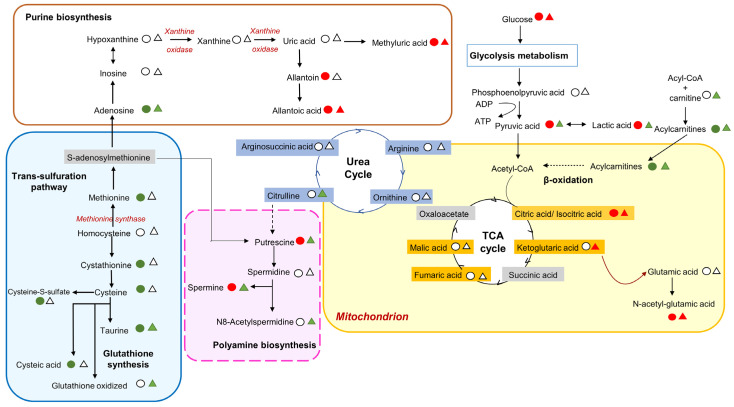
Modulated metabolic pathways in rabbit plasma post-ocular exposure to SM. Circles correspond to results from cohort 1 and triangles indicate the results from cohort 2. Green: increased metabolite levels in SM group compared to control group. Red: decreased metabolite levels in SM group compared to control group. White: metabolites that do not show any significant changes between SM and control groups. Grey box: not detected.

For example, the results showed an alteration of the sulfur-containing amino acids (SAAs) abundances, indicating a dysregulation of the trans-sulfuration pathway following ocular exposure to SM (Table 1 and Figure 3). In fact, taurine levels were upregulated on day 3 (FC = 1.59), day 8 (FC = 1.46), and day 15 (FC = 1.32) in cohort 1, and on day 3 (FC = 1.71) in cohort 2.

Our results also indicated the significant accumulation in cohort 1 of cystathionine on day 8 and day 15 (FC = 1.45 and FC = 1.76, respectively); methionine (FC = 1.26), cysteine (FC = 1.37), and cysteic acid (FC = 1.81) on day 15; and cysteine-S-sulfate on day 21 (FC = 1.54) and day 28 (FC = 1.50) post-exposure to SM (Table 1). SAAs have antioxidant properties and are produced following the accumulation of ROS. These observations are in line with the marked oxidative stress via the overproduction of ROS associated with SM-exposure [46]. Particularly, taurine is an antioxidant with osmotic properties that contributes to limiting the effect of ROS-induced plasma membrane permeability [39]. Most of the SAAs increased between day 3 and day 15 in the plasma of rabbits exposed to SM, suggesting antioxidant and anti-inflammatory activities in an attempt to reduce inflammation-related damage. Likewise, N-acetyl-cysteine (NAC), which is an acetylated form of cysteine, has been described as a potent antioxidant capable of scavenging free radicals and improving epithelial healing of injured animal eyes [47].

Of note, we did not detect any conjugates of SM with cysteine (Cys), N-acetyl-cysteine (NAC), and guanine (Gua), either in the plasma of unexposed or ocularly SM-exposed animals at any time (Figure S2). Figure S2 presents representative results obtained for these adducts at days 1, 8, and 21 post-exposure. This is in sharp contrast with what we observed previously after the cutaneous exposure of mice to CEES, where a maximum plasma concentration of the Cyst, NAC, Gua, and glutathione adducts was observed after one day [31]. Similarly, the diffusion of SM through the skin to the internal organs was evidenced in mice by the presence of DNA adducts at as early as 6 h after exposure [12,13]. Thus, in contrast to cutaneous exposure, it seems that SM and/or SM adducts do not diffuse from the eye to the blood.

On the other hand, amino acids (AAs) belonging to the phenylalanine, tyrosine, and tryptophan biosynthesis pathways were modulated in plasma after ocular exposure to SM. As previously mentioned, our results showed the accumulation of 3,4-dihydroxy-phenylalanine (Dopa) on day 1 (FC = 11.86) and day 8 (FC = 3.27) in cohorts 1 and 2, respectively (Table 1). Likewise, tyrosine decreased up to day 8 in cohort 1 and at day 28 in cohort 2, while N-acetyl-tyrosine increased on day 21 (FC = 1.98) in cohort 1 (Table 1). The dysregulation of the tryptophan pathway was observed in the plasma of exposed rabbits when compared to controls (Table 1). Results from cohort 1 showed that the levels of N-acetyl-tryptophan and kynurenine levels were decreased on day 3, while those of kynurenic acid decreased between 1 and 3 days after SM exposure (Table 1). Inversely, hydroxykynurenine abundances decreased in cohort 1 and increased in cohort 2 between day 3 and day 8 post-exposure. Additionally, we noted the increase in the abundances of quinaldic acid on day 1 (FC = 1.76) and serotonin on day 1 (FC = 2.09) and day 28 (FC = 3.23).

Quinolinic acid was downregulated specifically in cohort 2 on day 1 (FC = 0.39) post-exposure to SM (Table 1). Tryptophan can be metabolized following two major pathways, as follows: the kynurenine pathway in both host immune cells and intestinal cells and the serotonin pathway performed by enteroendocrine cells. The present results suggest preferential tryptophan oxidation via the kynurenine pathway as a response to oxidative stress [48].

Kynurenine-related metabolites can induce the activation of the Aryl hydrocarbon Receptor (AhR) that is involved in the differentiation of T cells and the antioxidant response [49].

In addition, purine biosynthesis was significantly altered in both cohorts after exposure to SM, especially with the downregulation of allantoic acid on day 8 in cohort 1 and on day 1 in cohort 2. Allantoic acid is a powerful antioxidant that may be intensively consumed in situations of acute inflammation. We also noted the similar downregulation of 1-methyluric acid and N-acetyl-asparagine on day 3 in cohort 1 and on day 28 in cohort 2 after exposure to SM (Table 1 and Figure 3). In fact, uric acid may represent a major endogenous defense against oxidative damage in the body by scavenging free radicals [50].

In addition, the dysregulation of energy metabolism was highlighted by significant changes in both cohorts; in particular, the inhibition of glycolysis or the TCA cycle in both cohorts with altered levels of isocitric/citric acid, lactic acid, pyruvic acid, and N-acetyl-glutamic acid in the first week post-exposure (Table 1 and Figure 3). Also, the levels of 3-isopropylmalic acid, ketoglutaric acid, and pyroglutamic acid l decreased over time in cohort 1 (Table 1). When glycolysis is unable to proceed, nicotinamide adenine dinucleotide (NADH) is mainly generated in the Krebs cycle from alternative substrates. It has been demonstrated that the presence of hydrogen peroxide (H2O2) inhibited α-ketoglutarate dehydrogenase (α-KGDH), which is correlated with a decrease in the nicotinamide adenine dinucleotide phosphate (NADPH) level, suggesting that the inhibition of this enzyme may be a critical factor in limiting the NADH production in the Krebs cycle during oxidative stress [51]. Moreover, α-KGDH has been described as an important regulatory site in the mitochondrial metabolism and a crucial target of ROS in cells, playing a key role in the bioenergetic deficit that occurs in oxidative stress [52]. In addition, 3-hydroxy-3-methylbutyric acid was unregulated at day 15 (FC = 1.65) and day 8 (FC = 6.35) in cohorts 1 and 2, respectively (Table 1), while 3-hydroxybutyric acid was upregulated at day 15 (FC = 1.40) only in cohort 2 (Table 1). Indeed, acetoacetate acid and 3-hydroxybutyrate were upregulated in the serum of SM-exposed patients [53]; these ketones are important byproducts of fatty acid oxidation in the liver.

Additionally, we also noticed a significant change in many circulating fatty acids in the plasma of both cohorts after SM exposure (Table 1). Excessive or insufficient lipogenesis has been reported to be associated with aberrations in lipid homoeostasis, potentially leading to pathological consequences such as fatty liver, autoimmune diseases, and neurodegenerative diseases [54].

Altogether, our results underlined the impairment of amino acid metabolism as the main oxidative stress-impacted metabolic pathways in plasma and bioenergetic metabolism, indicating mitochondrial dysregulation and dysfunction [55]. Overall, these manifested metabolic changes in plasma can be directly related to the local ocular injury caused by SM exposure.

#### 3.4.2. Metabolites Potentially Associated with Ocular Neovascularization

The clinical observations have highlighted increased neovascularization after exposure to SM (Figure S1). As previously described, both the early and chronic states have been shown to be associated with inflammation and neovascularization, leading to potential irreversible corneal damage and blindness depending on SM dose [32].

Our results showed the significant accumulation of adenosine in rabbit plasma on day 15 (FC = 2.06) and day 28 (FC = 1.85) in cohort 1, and on day 8 (FC = 3.63) in cohort 2 (Table 1). A recent study showed that 24 h after exposure of human epithelium model cells to SM, several metabolites belonging to the adenosine metabolic pathway (adenosine, inosine, adenine, and hypoxanthine) were significantly up or downregulated [56]. Adenosine could modulate a variety of cellular functions by interacting with specific adenosine receptors (AdoR) on the cell surface. Interestingly, the AdoR antagonist xanthine amine congener, which is potent but non-selective, was able to reduce the extent of neovascularization [57].

Moreover, adenosine has also been shown to cause vasodilation by contributing to the improved oxygenation of tissues, stimulating the proliferation of a variety of cell types including capillary endothelial cells, and promoting angiogenesis. The ATP-sensitive potassium currents are generated primarily in the capillary portion of retinal capillary/arteriolar segments; these capillaries are redox-sensitive and their function depends on the cellular production of oxidants during the catabolism of polyamines [58]. Thus, the vasodilator response to adenosine in the retinal capillaries proved dependent on polyamine-driven oxidation within the endothelial cells [59].

Consistently, we found that polyamine biosynthesis was particularly affected in the plasma of SM-exposed rabbits. The putrescine, spermine, and N8-acetyl-spermidine levels were strongly upregulated (FC = 2.62, FC = 3.52 and FC = 4.17, respectively) on day 8 post-SM-exposure in cohort 2 (Table 1 and Figure 3). In cohort 1, the result showed that only putrescine was significantly dysregulated on day 28 (FC = 0.81, *p*-value = 0.004) (Table 1 and Figure 3). As discussed above, such a different behavior of polyamine levels between the two cohorts might be due to inter-individual variability. Interestingly, previous works found that the concentration of spermine was reduced in the aqueous humor [60] and plasma [61] of patients with primary open-angle glaucoma (POAG). Polyamines are involved in the functioning, transformation, growth, and differentiation of cells; these molecules possess anti-apoptotic and neuroprotective properties [62]. The inhibition of their biosynthesis, or the administration of polyamine analogs that mimic natural polyamines but that have altered function, blocks cell proliferation, which could potentially be useful in treatments for diseases in which there is an excessive proliferation of cells [63]. Periocular injection of polyamine analogs was shown to inhibit retinal or choroidal neovascularization and a single injection provides inhibitory activity for at least 2 to 3 weeks, which could provide the basis for a feasible treatment for clinical trials [64].

Otherwise, we noted the specific upregulation in both cohorts of circulating carnitine, as well as acetyl-carnitine, propionyl-carnitine, and butyryl-carnitine, by comparing the SM-exposed group with the control group during the first two weeks post-exposure (Table 1 and Figure 2B).

The release of small acyl-carnitines from mitochondria into the blood stream could result from the incomplete β-oxidation of fatty acids [65]. Long-chain acylcarnitines were not detected in our metabolomics signature, which does not argue in favor of a defect of mitochondrial oxidation. Buisset et al. reported that the absence of long-chain acylcarnitines and an increase in the concentrations of amino acids together with that of short-chain acylcarnitines in the aqueous humor of patients with glaucoma are compatible with a deficient amino acid metabolism in cells drained by the aqueous humor [60].

L-carnitine and short-chain acyl-carnitines were reviewed as potential metabolic biomarkers in the plasma/serum, aqueous/vitreous humor, and tears of patients with common ocular diseases, such as age-related macular degeneration, diabetic retinopathy, retinopathy of prematurity, central retinal vein occlusion, primary open-angle glaucoma, rhegmatogenous retinal detachment, and dry eye syndrome [66]. Free carnitine and short-chain fatty acids acylcarnitines (C2, C3, and C4) levels were higher in the aqueous humor [60] and plasma [61] of patients with POAG than in controls. Carnitine has been shown to have neuroprotective, antiapoptotic, and antioxidant properties, so that increasing the carnitine concentration may protect retinal cells against disease-related stress such as that due to ROS production [67]. The alteration of acyl-carnitines was associated with the presence of ischemia and neovascularization in ocular diseases [68].

Altogether, these results demonstrated the significant over expression of adenosine, polyamines, carnitine, and short-chain acyl-carnitines levels in plasma as candidate biomarkers of the neovascularization occurred following ocular exposure to SM.

#### 3.4.3. Additional Systemic Effects Induced by SM Ocular Exposure and Potentially Associated with Microbiota

Further metabolic perturbations were highlighted in plasma, resulting from systemic effects of SM toxicity after ocular exposure. In fact, we found that the abundance of 5-hydroxyindole-acetic acid was strongly upregulated on day 8 (FC = 10.61) and day 3 (FC = 3.36) in cohorts 1 and 2, respectively (Table 1). Indole-acetic acid and indole-acrylic acid were upregulated on day 8 and day 15 in cohorts 1 and 2, respectively (Table 1). The accumulation of indole species might indicate the regulation of immune responses, through the activation of AhR [69]. The above described perturbation of the serotonin, kynurenine, and indole metabolic pathways reflects the microbial regulation of circulating tryptophan [48], which may be related to dysbiosis and neurological disorders consecutively to SM exposure. Of note, further specific changes were specifically highlighted in cohort 2, indicating gut microbiota changes and/or liver dysfunction.

We also noted the upregulation of several bile acids (such as cholic acid, glycocholic acid, glycodoxycholic acid, and ursodeoxycholic acid) at day 15 (Table 1), while trimethylamine-N-oxide (TMAO), phosphorylethanolamine, and acetylcholine were similarly accumulated at day 8. Particularly, TMAO was associated with cardiovascular diseases through the impairment of vascular endothelial function via superoxide-driven oxidative stress [70]. Since it is mainly derived from the gut, TMAO concentration depends on the type and amount of consumed dietary nutrients. Given their dietary origin, the dysregulation of these metabolites as candidate biomarkers needs to be confirmed in further cohorts.

## 4. Conclusions

The untargeted metabolomics approach performed in this study reveals significant perturbations of the metabolite profiles in the plasma of rabbits after ocular exposure to SM and provides useful information regarding the metabolic pathways that are dysregulated and associated with the different phases of ocular damage. Despite a certain inter-individual variability, disturbances in energy metabolism, amino acid metabolism, and purine and polyamine biosynthesis were consistently observed in the two cohorts studied and were related to a strong oxidative stress occurring post-exposure.

Taurine and Dopa, were among the significantly and specifically altered metabolites and may constitute useful biomarkers. Afterwards, further metabolic changes occurred from week 2 post-exposure, which could be related to the evolution of observed ocular lesions and could essentially be involved in the dysregulations of the purine and polyamine biosynthesis, tryptophan, and energy metabolism pathways. Metabolic changes in plasma for adenosine, polyamines, and acyl-carnitines potentially reflect ocular neovascularization. Meanwhile, the inability to detect SM adducts (Cyc-SM, NAC-SM, and Gua-SM) in rabbit plasma might underline the absence of diffusion from eye to blood.

Overall, the present work demonstrated clearly the direct impact of ocular exposure to SM on the plasma metabolic profiles and highlighted the specific associated metabolic pathways that could be of interest for the development of further treatment strategies. Performing untargeted metabolomics analysis of rabbit tears could represent an interesting perspective to obtain additional information on SM toxicity and as a source of less invasive biomarkers. Further studies using other omics approaches may be of interest to complement the metabolomics data and to provide a more comprehensive understanding of the complex biological effects of SM after ocular exposure.

## Figures and Tables

**Table 1:** Metabolic changes observed in plasma after SM exposure in the first and second cohorts.

			Cohort 1			Cohort 2	
	Metabolite	Time Point ^1^	Fold Change (SM/CTL) ^2^	*p*-Value	Time Point ^1^	Fold Change (SM/CTL) ^2^	*p*-Value
Transulfuration pathway	Cystathionine	day 8	1.45	0.028			ns
		day 15	1.76	0.021			
	Cysteic acid	day 15	1.81	0.033			ns
	Cysteine	day 15	1.37	0.028			ns
	Cysteine-S-sulphate	day 21	1.54	0.028			ns
		day 28	1.50	0.048			
	Glutathione oxidized			ns	day 3	3.10	0.042
	Methionine	day 15	1.26	0.048			ns
	Methionine sulfoxide			ns	day 8	0.59	0.028
	N-Acetyl-methionine	day 3	0.75	0.048			ns
	Cysteine-S-sulphate	day 21	1.54	0.028			ns
	Taurine	day 3	1.59	0.016	day 3	1.71	0.012
		day 8	1.46	0.016			
		day 15	1.32	0.048			
Catabolism of branched-chain amino acids	3-Hydroxy-3-methylbutyric acid	day 15	1.65	0.020	day 8	6.35	0.042
	3-Hydroxy-butyric acid			ns	day 15	1.40	0.048
	Isoleucine	day 3	0.75	0.016	ns		
	Isovaleric acid			ns	day 15	1.76	0.004
	Lysine			ns	day 28	0.69	0.048
	N-acetyl-isoleucine	day 3	0.63	0.048	day 21	1.85	0.016
	N6-acetyl-lysine	day 15	1.45	0.028			ns
	N6,N6,N6-Trimethyl-lysine			ns	day 28	0.65	0.012
	Valine	day 3	0.73	0.033	day 28	0.68	0.012
Phenylalanine, tyrosine, and tryptophan metabolic pathway	3,4-Dihydroxy-phenylalanine (Dopa)	day 1	11.86	0.024	day 8	3.27	0.006
	3-Hydroxy-cinnamic acid (Coumaric acid)	day 3	0.69	0.028	day 1	0.54	0.048
	α-Hydroxy-hippuric acid	day 3	0.56	0.016	day 21	0.51	0.024
	4-Hydroxy-phenylglycine	day 21	1.91	0.048	day 15	0.41	0.048
	5-Hydroxy-indoleacetic acid	day 8	10.61	0.006	day 3	3.36	0.038
	Hydroxy-kynurenine	day 3	0.77	0.048	day 3	2.54	0.038
		day 8	0.65	0.020	day 8	3.70	0.006
	Indoleacetic acid	day 8	2.63	0.028			ns
	Indole-3-propionic acid	day 1	0.62	0.024			ns
		day 3	0.51	0.041			
	Kynurenine	day 3	0.70	0.028			ns
	N-acetyl-tryptophan	day 3	0.69	0.048			ns
	N-acetyl-tyrosine	day 21	1.98	0.008			ns
	2-Phenylglycine	day 28	1.30	0.016	day 8	0.64	0.016
	Quinaldic acid	day 1	1.76	0.024			ns
	Quinolinic acid		ns	0.008	day 1	0.39	0.048
	Serotonine	day 1	2.09	0.028			ns
		day 28	3.23	0.028			
	Tryptophan	day 3	0.79	0.028	day 28	0.83 *	0.048
	Tyrosine	day 3	0.71	0.048	day 28	0.69	0.024
		day 8	0.70	0.016			ns
Polyamines biosynthesis	N8-Acetyl-spermidine			ns	day 8	4.17	0.024
	Putrescine	day 28	0.81 *	0.004	day 8	2.62	0.048
	Spermine	day 1	0.93 *	0.048	day 8	3.52	0.024
Purines biosynthesis	Adenosine	day 15	2.06	0.034	day 8	3.63	0.042
		day 28	1.85	0.028			
	Allantoic acid	day 8	0.54	0.033	day 1	0.54	0.048
	Allantoin	day 3	0.57	0.028			ns
	Guanosine	day 21	0.49	0.048			ns
	Guanine			ns	day 8	0.55	0.004
	Methyluric acid	day 3	0.72	0.048	day 28	0.67	0.048
	7-Methyl- xanthine			ns	day 8	3.65	0.042
	N-acetyl-asparagine	day 3	0.45	0.028	day 28	0.52	0.012
	2-O-methyl-guanosine	day 1	0.86 *	0.042			ns
	2-O-methyl-inosine			ns	day8	6.16	0.048
	Proline	day 3	0.63	0.048			ns
Glycolysis pathway	Fructose (ketoses)			ns	day 8	0.50	0.048
	Gluconic acid			ns	day 28	1.63	0.024
	Glucose 1-phosphate (hexose-phosphate)						
	Lactic acid	day 8	0.67	0.028	day 15	1.64	0.048
		day 28	0.56	0.048			
	Pyruvic acid	day 1	0.67	0.012	day 21	1.57	0.008
		day 3	0.60	0.048			
	Glucose (hexoses)	day 3	0.70	0.027	day 8	0.50	0.028
TCA cycle	3-Isopropyl- malic acid	day 8	0.53	0.028			ns
		day21	0.47	0.048			
	Ketoglutaric acid	day 1	0.75	0.006			ns
	N-acetyl-glutamic acid	day 8	0.67	0.014	day 1	0.51	0.028
	Isocitric acid/Citric acid	day 1	0.70	0.012	day 1	0.52	0.028
		day 8	0.80 *	0.016			
		day 21	0.84 *	0.048			
	Pyroglutamic acid	day 3	0.51	0.008			ns
Choline-Trimethylamine N-Oxide (TMAO) pathway	Acetylcholine	day 3	0.75	0.028	day 8	4.18	0.024
	Phosphorylethanolamine			ns	day 3	1.66	0.024
	Trimethylamine oxide			ns	day 8	3.48	0.042
Carnitines	Acetylcarnitine			ns	day 8	5.12	0.048
	Butyrylcarnitine	day 15	1.07 *	0.048	day 8	4.12	0.024
	Carnitine			ns	day 3	1.68	0.024
					day 8	3.10	0.012
	Propionyl-carnitine			ns	day 8	6.23	0.024
Fatty acids	Arachidic acid			ns	day 28	2.03	0.048
	Caproic acid	day 15	1.36	0.028			
	Cholic acid			ns	day 15	4.13	0.004
	Dodecanedioic acid			ns	day 1	0.57	0.048
	Eicosapentaenoic acid	day 28	1.37	0.016	day 21	2.59	0.016
	Glycocholic acid			ns	day 15	6.55	0.008
	Glycochenodeoxycholic acid			ns	day 15	4.43	0.008
	Hydroxy-eicosatetraenoic acid	day 3	0.38	0.028	day 3	2.06	0.041
	Hydroxy- stearic acid	day 21	4.29	0.028			ns
	Lithocholic acid			ns	day 1	0.26	0.016
	Mesaconic acid			ns	day 21	1.57	0.048
	Myristic acid			ns	day 21	4.51	0.028
	Monomethyl adipic acid	day 15	1.81	0.008			ns
	Monomethyl glutaric acid			ns	day 21	2.11	0.016
	Perillic acid			ns	day 15	5.64	0.028
	Deoxycholic acid			ns	day 15	3.24	0.016
	Elaidic acid			ns	day 21	2.49	0.028

^1^ Indicates that the time point at which the SM-exposed group was compared with the control group was significantly different. ^2^ Fold change are ratios of mean abundances in the SM-exposed group to mean abundances in the controls. Asterisk indicates significant change of metabolite abundances between control group and SM-exposed group (*p* < 0.05), but a low fold change (FC > 0.8; <1.2). *p*-values have been obtained from a non-parametric Wilcoxon Mann–Whitney test (*p* < 0.05) between the SM group and the control group at the corresponding time point. ns: not significant between control and SM group over time.

## Data Availability

The data presented in this study are available on request from the corresponding author. The data are not publicly available due to privacy.

## Supplementary Materials

The following supporting information can be downloaded at: https://www.mdpi.com/article/10.3390/metabo14070349/s1. Figure S1: Temporal evolution of eye neovascularization in control and SM-exposed rabbits from cohorts 1 and 2; Figure S2: Targeted analysis using HPLC–MS/MS of 2-hydroxyethyl-thioethyl-S-cysteine (Cys-SM), 2-hydroxyethyl-thioethyl-S-N-acetylcysteine (NAC-SM), and 2-hydroxyethyl-thioethyl-N7-guanine (Gua-SM) in the plasma of rabbits ocularly exposed to SM; Table S1: Internal and external chemical standards used for LC–HRMS analysis; Table S2: List of metabolites annotated in plasma of the control and SM-exposed groups; Table S3: List of significant metabolites between the control and SM-exposed groups at different time points in both C01 and C02 cohorts.
