# Supplementary material for: Metabolomics Analysis of Rabbit Plasma after Ocular Exposure to Vapors of Sulfur Mustard
Source: Metabolites. 2024 Jun 21;14(7):349. doi: 10.3390/metabo14070349 (PMC11279318; doi:10.3390/metabo14070349)
Supplement: Supplementary file 1 [file metabolites-14-00349-s001.zip › Bouhlel et al-Supporting Information - Copy.pdf]

Supporting Information

Metabolomics analysis of rabbit plasma after ocular exposure to vapors of sulfur mustard

Jihene Bouhlel [1], Fanny Caffin [2], Fanny Gros-Désormeaux [2], Thierry Douki [3], Jean-François Benoist [1, 4], Florence A. Castelli [1], Emeline Chu-Van [1], Christophe Piérard [2], Christophe Junot [1], François Fenaille [1,*]

[1] Université Paris-Saclay, CEA, INRAE, Département Médicaments et Technologies pour la Santé (DMTS), SPI, MetaboHUB-IDF, 91191 Gif-sur-Yvette, France
[2] Institut de Recherche Biomédicale des Armées (IRBA), 91223 Brétigny-sur-Orge, France
[3] Université Grenoble Alpes, CEA, CNRS, IRIG, SyMMES, F-38000, Grenoble, France
[4] Biochemistry Laboratory, APHP, Hôpital Universitaire Necker Enfants Malades, Paris, France
* Correspondence: francois.fenaille@cea.fr

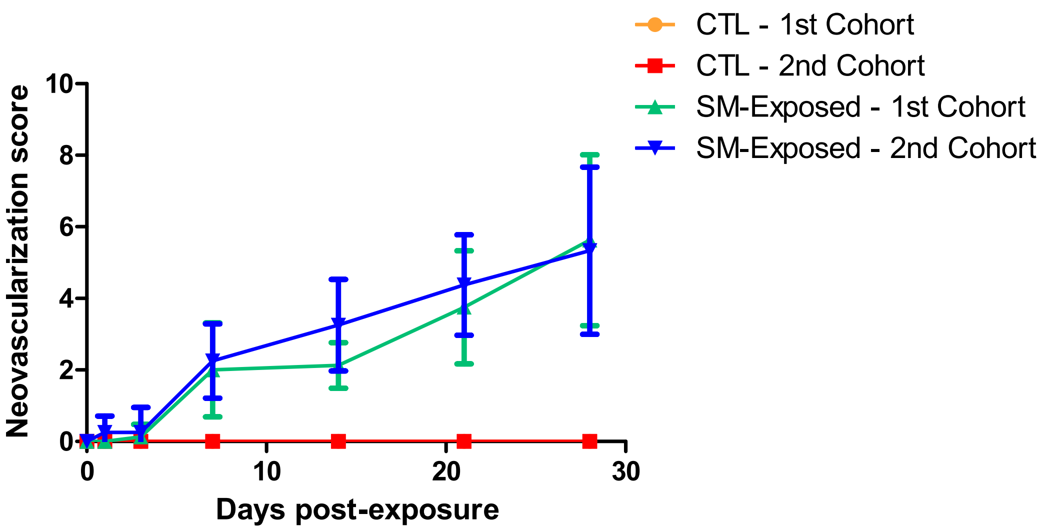

Figure S1. Temporal evolution of eye neovascularization in control and SM-exposed rabbits from cohorts 1 and 2. Neovascularization score (as a percentage) was obtained from the ratio between the length of the neovessel and the radius of the cornea (divided into 4 quarters), both were measured using ImageJ Fiji software. Scores (percentage) ranged from 0 (no neovascularization) to 4 (length greater than 75% of the radius of the cornea). The score for each quarter were then summed to obtain a single value per animal per day.

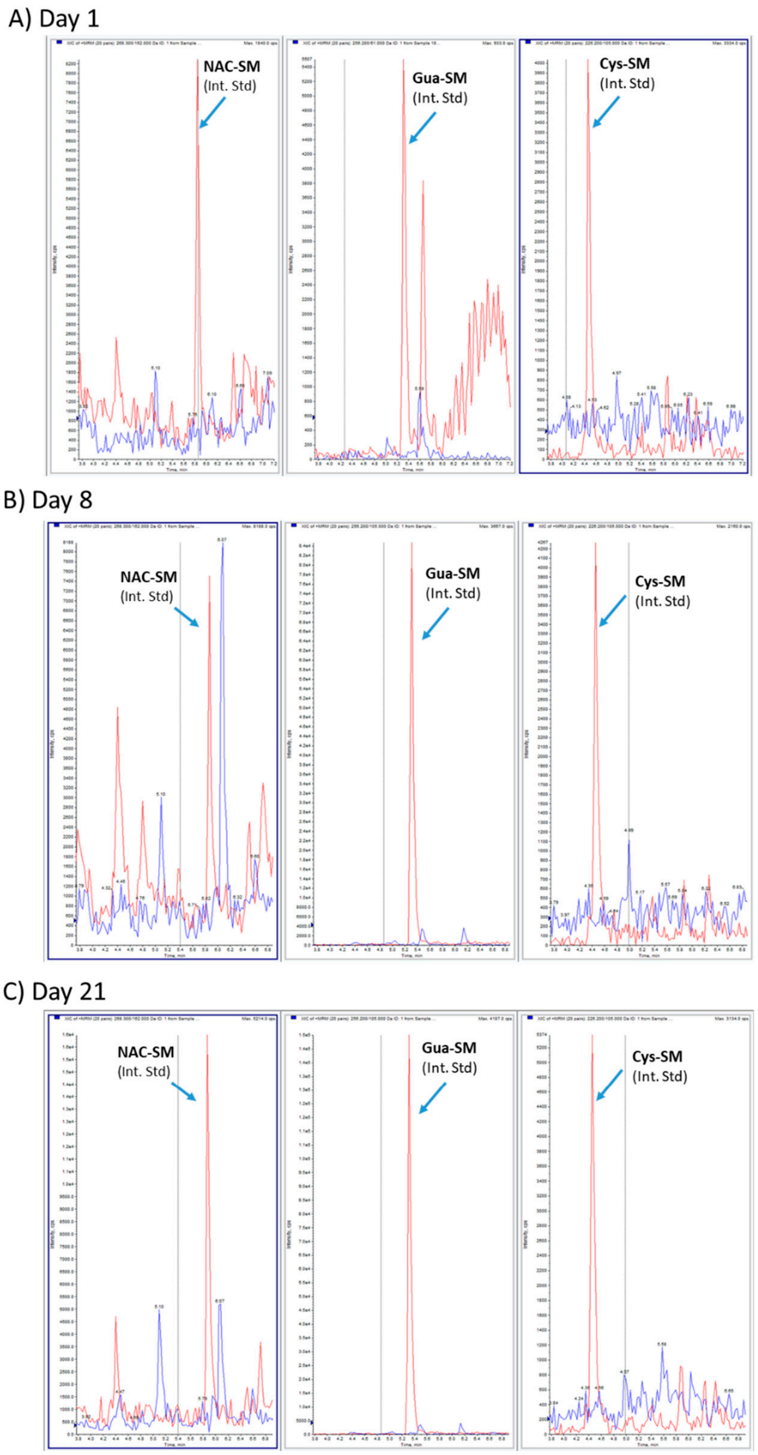

Figure S2. Targeted analysis using HPLC-MS/MS of 2-hydroxyethyl-thioethyl-S-cysteine (Cys-SM), 2-hydroxyethyl-thioethyl-S-N-acetylcysteine (NAC-SM) and 2-hydroxyethyl-thioethyl-N7-guanine (Gua-SM) in the plasma of rabbits ocularly exposed to SM. Presented are representative chromatograms obtained at A) day 1, B) day 8 (B) and C) day 21 post-exposure. Blue and red traces correspond to

those of unlabeled and labeled species, respectively. The following multiple reaction monitoring (MRM) transitions 226 -> 105, 268 -> 162 and 256 -> 105 were monitored for Cys-SM, NAC-SM and Gua-SM, respectively. The retention times of Cys-SM, NAC-SM and Gua-SM were 4.5 min, 5.9 min and 5.3 min under the HPLC conditions used, respectively.
